# Sodium-Glucose Cotransporter-2 (SGLT2) Inhibitors and Risk of Heart Failure Hospitalization in Type 2 Diabetes: A Systematic Review and Meta-Analysis of Randomized Controlled Trials

**DOI:** 10.7759/cureus.96456

**Published:** 2025-11-09

**Authors:** Iman Joher, Shivam Singla, Umama Shakeel Ahmed, Bhavna Singla, Saniya Amir, Mehak G Mastoi, Sunita Kumawat, Rishail Khalid, Faiza Choudhry, Syed Ali Abbas Rahat, Asim Iqbal

**Affiliations:** 1 Internal Medicine, Rawalpindi Medical University, Rawalpindi, PAK; 2 Internal Medicine, TidalHealth Peninsula Regional, Salisbury, USA; 3 General Surgery, South City Hospital, Karachi, PAK; 4 Internal Medicine, Erie County Medical Center Health Campus, Buffalo, USA; 5 Accident and Emergency, Liaquat National Hospital and Medical College, Karachi, PAK; 6 Geriatrics, Montefiore Medical Center Wakefield Campus, New York, USA; 7 Internal Medicine, Interfaith Medical Center, New York, USA; 8 Internal Medicine, St. Francis Medical Center, Lynwood, USA; 9 General Surgery, Rashid Latif Medical College, Lahore, PAK; 10 Internal Medicine, Peoples University of Medical and Health Sciences for Women, Nawabshah, PAK; 11 General Surgery, Osh State University, Osh, KGZ; 12 Internal Medicine, Lahore Medical and Dental College, Lahore, PAK

**Keywords:** canagliflozin, cardiovascular outcomes, dapagliflozin, empagliflozin, ertugliflozin, heart failure hospitalization, meta-analysis, sglt2 inhibitors, sotagliflozin, type 2 diabetes mellitus

## Abstract

Sodium-glucose cotransporter-2 (SGLT2) inhibitors have emerged as a transformative therapy in type 2 diabetes mellitus (T2DM), offering benefits that extend beyond glycemic control. We conducted a meta-analysis of six large randomized controlled trials (RCTs), enrolling more than 47,000 patients with T2DM and varying risks of cardiovascular disease (CVD) and chronic kidney disease (CKD), to evaluate the effect of SGLT2 inhibitors on hospitalization for heart failure (HHF). Across a mean follow-up ranging from 1.3 to 4.2 years, SGLT2 inhibitors were associated with a 28% relative risk reduction in HHF compared with placebo or standard care. This benefit was consistent across most agents, including empagliflozin, canagliflozin, dapagliflozin, and sotagliflozin, while ertugliflozin showed a nonsignificant trend in the same direction. Subgroup analyses confirmed benefits in patients with established atherosclerotic CVD as well as those with CKD, underscoring the broad applicability of this therapy. The results demonstrate that SGLT2 inhibitors confer clinically meaningful cardiorenal protection that is recognized to occur through mechanisms largely independent of glucose lowering, reinforcing their role as cornerstone agents in the management of T2DM. These findings highlight the importance of prioritizing SGLT2 inhibitors in contemporary diabetes care to reduce the global burden of heart failure (HF).

## Introduction and background

Type 2 diabetes mellitus (T2DM) is a growing global health challenge, currently impacting more than 500 million individuals worldwide, with numbers expected to rise significantly in the coming decades. Beyond microvascular complications such as nephropathy and retinopathy, T2DM is strongly associated with macrovascular disease, particularly heart failure (HF) [1]. Patients with T2DM face a two- to five-fold increased risk of developing HF compared to non-diabetic individuals, and hospitalization for HF (HHF) is among the leading causes of morbidity, mortality, and healthcare expenditure in this population [2].

Despite advances in standard antidiabetic and cardiovascular therapies, optimal strategies to reduce HHF risk in T2DM remain limited. While some traditional glucose-lowering agents, such as insulin and sulfonylureas, have demonstrated neutral or even adverse effects on cardiovascular outcomes, others, like metformin and thiazolidinediones, have shown potential cardiovascular benefit [3]. This underscores the need for agents that provide both glycemic control and consistent cardiovascular protection.

Sodium-glucose cotransporter-2 (SGLT2) inhibitors have emerged as a transformative drug class in the management of T2DM. By inhibiting renal glucose reabsorption, these agents improve glycemic control while also exerting favorable effects on blood pressure, weight, and renal hemodynamics [4]. Importantly, large cardiovascular outcome trials (CVOTs) such as EMPA-REG OUTCOME [5], CANVAS [6], DECLARE-TIMI 58 [7], CREDENCE [8], VERTIS-CV [9], and SCORED [10] have consistently demonstrated that SGLT2 inhibitors significantly reduce the risk of HHF, irrespective of baseline cardiovascular status.

While these findings have led to widespread adoption of SGLT2 inhibitors in clinical practice and guideline recommendations, uncertainties remain regarding the consistency of benefit across different agents within the class. Individual CVOTs have varied in study design, patient population, and outcome definitions, making it challenging to draw direct comparisons. Moreover, the relative magnitude of effect between empagliflozin, dapagliflozin, canagliflozin, ertugliflozin, and sotagliflozin has not been fully clarified.

This systematic review and meta-analysis aims to synthesize evidence from randomized controlled trials (RCTs) to evaluate the efficacy of SGLT2 inhibitors in reducing hospitalization for heart failure among patients with T2DM. By conducting subgroup analyses by drug, this study seeks to provide a clearer understanding of class effects versus drug-specific benefits, offering clinicians and policymakers robust evidence to guide treatment decisions.

## Review

Materials and methods

Study Design and Protocol

This systematic review and meta-analysis was conducted in accordance with the Preferred Reporting Items for Systematic Reviews and Meta-Analyses (PRISMA) guidelines [11]. The protocol was developed a priori and followed Cochrane Collaboration methodology to ensure methodological rigor and transparency. Although the protocol was not registered in PROSPERO or other databases, all stages - from study selection and data extraction to quality assessment and statistical analysis - were performed according to predefined criteria established before data collection, thereby minimizing bias and maintaining reproducibility.

Eligibility Criteria (PICO Framework)

We applied the PICO (Population/Patient/Problem, Intervention, Comparison/Control, and Outcome) framework [12] to clearly define the eligibility criteria for this review. The population included adults with T2DM, with or without established cardiovascular disease (CVD) or chronic kidney disease (CKD). The intervention of interest was treatment with SGLT2 inhibitors, specifically empagliflozin, dapagliflozin, canagliflozin, ertugliflozin, and sotagliflozin. The comparator was placebo or standard of care, which generally included background therapies consistent with guideline-directed medical treatment for diabetes and cardiovascular risk reduction (e.g., metformin, statins, angiotensin converting enzyme (ACE) inhibitors/angiotensin receptor blockers (ARBs), and antiplatelet therapy, as applicable). The primary outcome was risk of HHF, while secondary outcomes, when reported, included cardiovascular and renal events. Only RCTs with a minimum follow-up of 12 months, published in peer-reviewed journals, and reporting HHF outcomes were considered eligible. We excluded observational studies, reviews, case reports, conference abstracts, and studies published in non-English languages.

Literature Search Strategy

A comprehensive electronic search of PubMed, Embase, the Cochrane Central Register of Controlled Trials (CENTRAL), and ClinicalTrials.gov was performed from database inception to September 2025. The search strategy combined controlled vocabulary (Medical Subject Headings (MeSH) terms) and free-text terms using Boolean operators to maximize sensitivity. Key terms included (“SGLT2 inhibitors” OR “empagliflozin” OR “dapagliflozin” OR “canagliflozin” OR “ertugliflozin” OR “sotagliflozin”) AND (“heart failure hospitalization” OR “hospitalization for heart failure”) AND (“type 2 diabetes” OR “T2DM”). Filters were applied to restrict results to RCTs published in English. In addition, the reference lists of included trials and relevant systematic reviews were manually screened to identify any further eligible studies not captured by the database search.

Study Selection

Two independent reviewers screened titles and abstracts for relevance, followed by a full-text review of potentially eligible studies. Discrepancies were resolved by discussion or consultation with a third reviewer. The PRISMA flow diagram was used to depict the study selection process, including reasons for exclusion.

Data Extraction and Management

A standardized data extraction form was used to collect study characteristics (author, year, population, intervention details, comparator, follow-up duration), number of participants, and HHF event rates. Where multiple treatment arms were present, relevant intervention groups were pooled according to Cochrane guidelines. Extracted data were cross-verified by two reviewers to minimize errors.

Risk of Bias Assessment

The methodological quality of included RCTs was evaluated independently by two reviewers using the Cochrane Risk of Bias (RoB 2.0) tool [13]. Domains assessed included random sequence generation, allocation concealment, blinding of participants and personnel, blinding of outcome assessment, completeness of outcome data, selective reporting, and other potential sources of bias. Disagreements were resolved through consensus.

Statistical Analysis

Meta-analysis was performed using Review Manager (RevMan) version 5.4.1. The treatment effect was expressed as the risk ratio (RR) with 95% confidence intervals (CIs) for dichotomous outcomes. A random-effects model (Mantel-Haenszel method) was applied to account for between-study heterogeneity. Statistical heterogeneity was assessed using the I² statistic, with thresholds of 25%, 50%, and 75% representing low, moderate, and high heterogeneity, respectively.

Subgroup and Sensitivity Analyses

Pre-specified subgroup analyses were performed according to individual SGLT2 inhibitors (empagliflozin, dapagliflozin, canagliflozin, ertugliflozin, and sotagliflozin). Sensitivity analyses were conducted by sequentially excluding individual studies to examine the robustness of results.

Assessment of Publication Bias

Publication bias was planned to be evaluated through visual inspection of funnel plots and statistical tests for asymmetry when ≥10 studies were available for an outcome. However, as fewer than 10 RCTs met the inclusion criteria, a formal assessment of publication bias was not feasible.

Results

Study Selection Process

**Figure 1:**
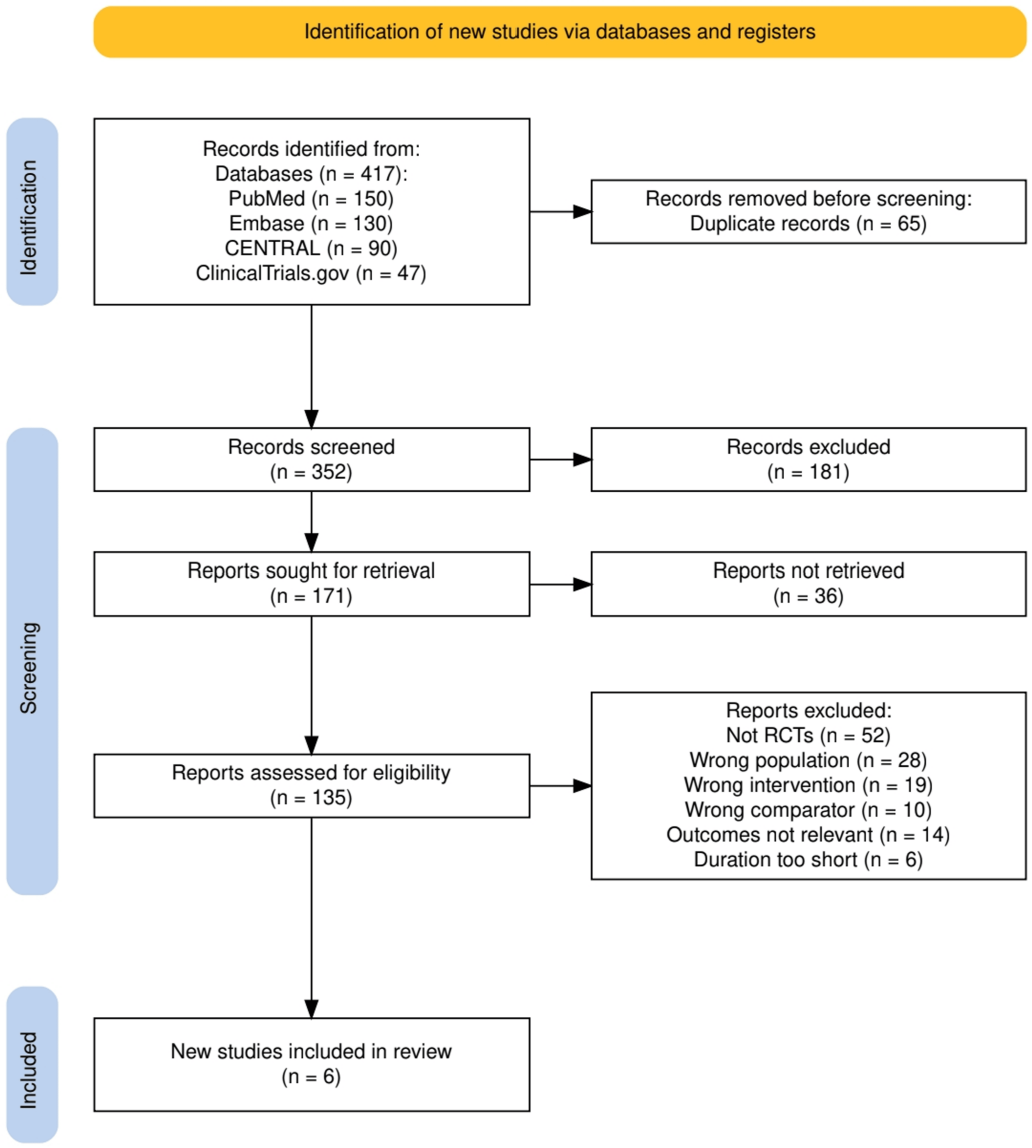
PRISMA flowchart depicting the study selection process PRISMA: Preferred Reporting Items for Systematic Reviews and Meta-Analyses

A total of 417 records were identified through database searches (PubMed, Embase, CENTRAL, and ClinicalTrials.gov). After removal of 65 duplicates, 352 records underwent title and abstract screening, of which 181 were excluded. The full text of 171 articles was sought, with 36 reports not retrieved. Of the remaining 135 articles assessed for eligibility, 129 were excluded for reasons including non-randomized design, wrong population, intervention, comparator, or outcome, and insufficient follow-up. Ultimately, six RCTs met the inclusion criteria and were synthesized in this meta-analysis. The detailed selection process is illustrated in Figure 1 (PRISMA flow diagram).

Characteristics of the Selected Studies

To provide a comprehensive overview of the evidence base, the key characteristics of the included RCTs are summarized in Table 1. These trials, conducted between 2015 and 2021, evaluated various SGLT2 inhibitors across diverse high-risk patient populations with T2DM. Differences in study design, population characteristics (such as the proportion of patients with established atherosclerotic CVD [ASCVD] or CKD), follow-up duration, and drug dosing strategies are highlighted. In all trials, SGLT2 inhibitors were compared with placebo administered on top of standard background therapy, which generally included guideline-directed treatments for diabetes and cardiovascular risk reduction (e.g., metformin, statins, ACE inhibitors/ARBs, and antiplatelet therapy, as applicable). Reporting the HHF events within each arm allows for direct comparison of the intervention versus the control groups. Additionally, the assessed risk of bias (RoB) judgments are included to inform the overall quality and reliability of the evidence.

**Table 1:** Characteristics of RCTs evaluating SGLT2 inhibitors and risk of HHF in patients with T2DM RCTs: randomized controlled trials; SGLT: sodium-glucose cotransporter; HHF: hospitalization for heart failure; T2DM: type 2 diabetes mellitus; Int: intervention; Ctrl: control; HF: heart failure; Hosp.: hospitalization; RoB: risk of bias; ASCVD: atherosclerotic cardiovascular disease; CV: cardiovascular; CKD: chronic kidney disease; RAS: renin–angiotensin system; eGFR: estimated glomerular filtration rate

Study (year)	Drug	Population	N (Int)	N (Ctrl)	HF Hosp. Events (Int)	HF Hosp. Events (Ctrl)	Follow-up (years)	Notes	RoB
EMPA-REG (2015) [5]	Empagliflozin	T2DM + established ASCVD	4687	2333	126	96	3.1	10/25 mg daily; pooled analysis; ASCVD pts	Low
CANVAS (2017) [6]	Canagliflozin	T2DM + high CV risk (65% ASCVD)	5795	4347	96	127	3.6	100/300 mg daily; integrated program	Low
DECLARE–TIMI 58 (2019) [7]	Dapagliflozin	T2DM + ASCVD or multiple risks	8582	8578	212	286	4.2	10 mg daily; broad population (40% ASCVD)	Low
VERTIS CV (2020) [9]	Ertugliflozin	T2DM + established ASCVD	5499	2747	212	124	3.5	5 mg or 15 mg daily; pooled analysis; ASCVD only	Low
CREDENCE (2019) [8]	Canagliflozin	T2DM + CKD (albuminuric, on RAS blockade)	2202	2199	61	100	2.62	100 mg daily; renal-focused trial	Low
SCORED (2021) [10]	Sotagliflozin	T2DM + CKD (eGFR 25–60, ± albuminuria)	5292	5292	118	170	1.3	Dual SGLT1/2; early termination; high-risk CKD	Some concerns

Quality Assessment

**Figure 2:**
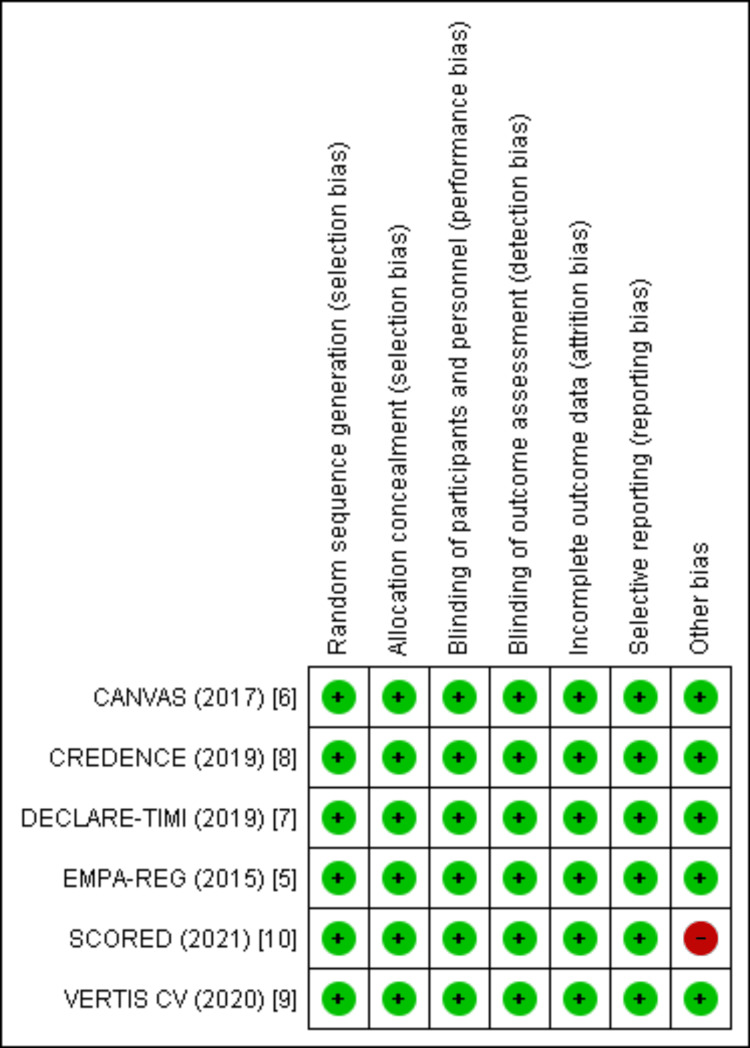
Study-level assessment of risk of bias across RCTs Risk-of-bias summary illustrating the judgment for each domain across the randomized controlled trials included in this review. Overall, all studies demonstrated a low risk in major domains, including random sequence generation, allocation concealment, blinding of participants/personnel, blinding of outcome assessment, incomplete outcome data, and selective reporting. One study (SCORED [10]) showed a high risk under the “other bias” domain. Trials assessed include EMPA-REG (2015) [5], CANVAS (2017) [6], DECLARE–TIMI 58 (2019) [7], CREDENCE (2019) [8], VERTIS CV (2020) [9], and SCORED (2021) [10] RCTs: randomized controlled trials

The methodological quality of the included RCTs was assessed using the Cochrane Risk of Bias tool. As illustrated in Figure 2, the overall risk of bias across studies was low. The majority of trials demonstrated adequate random sequence generation, allocation concealment, and blinding of participants, personnel, and outcome assessors. Incomplete outcome data and selective reporting were also judged as low risk in all studies. However, one trial was rated as having a high risk of “other bias,” primarily due to early termination and related methodological limitations. Despite this, the consistency of low risk across key domains suggests that the included evidence is of generally high methodological quality.

**Figure 3:**
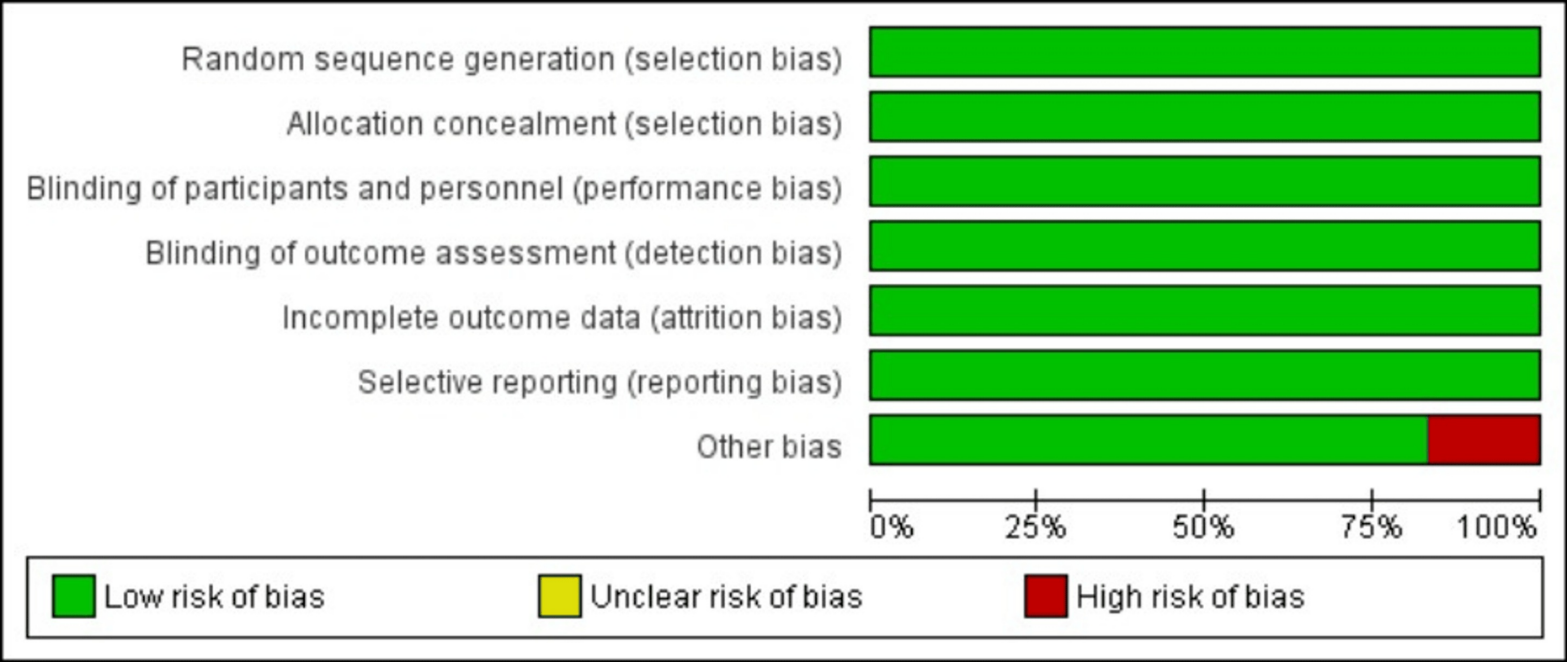
Overall risk of bias across included RCTs showing predominantly low risk, with a small proportion rated as high risk for other bias RCTs: randomized controlled trials

The study-level assessment is presented in Figure 3, which provides a detailed breakdown of individual judgments across all domains of bias for each included trial. All six trials were judged to have a low risk of bias in randomization, allocation concealment, blinding, and outcome reporting. Only the one trial was flagged for high risk under “other bias” due to premature termination, which may have influenced event rates and overall precision of results. Importantly, no study was identified as having unclear risk in any critical domain, reinforcing the overall robustness and reliability of the pooled evidence.

Primary Outcome: Hospitalization for Heart Failure (HHF)

**Figure 4:**
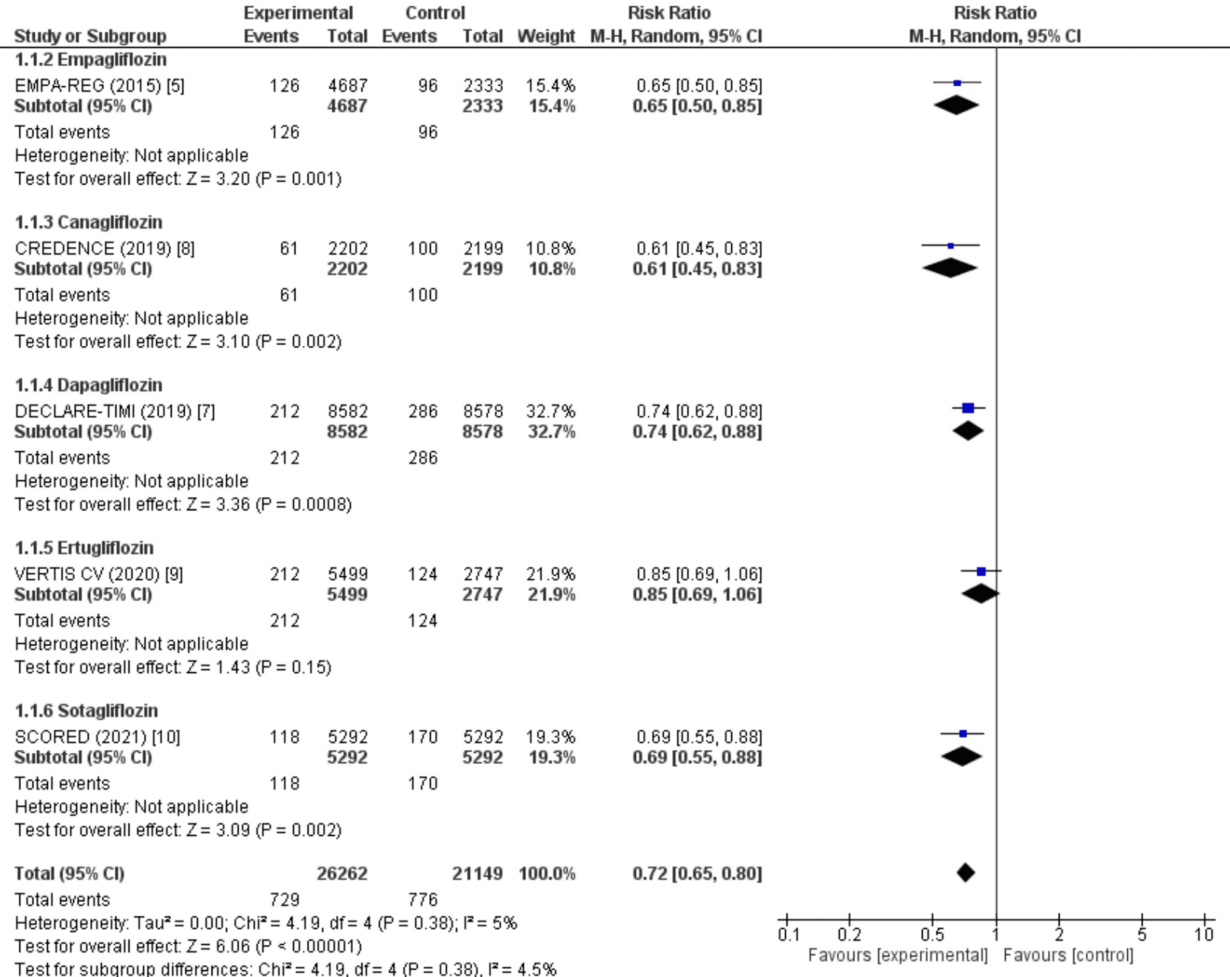
Effect of SGLT2 inhibitors on HHF in patients with T2DM Forest plot showing the comparative effect of SGLT2 inhibitors versus control on HHF among patients with T2DM. Subgroup labels (1.1.2–1.1.6) correspond to individual SGLT2 inhibitor trials: 1.1.2 – Empagliflozin (EMPA-REG [5]), 1.1.3 – Canagliflozin (CREDENCE [8]), 1.1.4 – Dapagliflozin (DECLARE–TIMI 58 [7]), 1.1.5 – Ertugliflozin (VERTIS CV [9]), and 1.1.6 – Sotagliflozin (SCORED [10]). Each horizontal line represents the 95% CI for the RR in individual studies, while the diamond indicates the pooled effect estimate with its CI SGLT: sodium-glucose cotransporter; HHF: hospitalization for heart failure; T2DM: type 2 diabetes mellitus; CI: confidence interval; RR: risk ratio

The pooled analysis of six RCTs, encompassing 47,411 participants (26,262 in the SGLT2 inhibitor group and 21,149 in the control group), demonstrated a significant reduction in the risk of HHF with SGLT2 inhibitors compared to placebo or standard care. The overall risk ratio (RR) was 0.72 (95% CI: 0.65-0.80, p<0.00001), indicating a 28% relative risk reduction. Statistical heterogeneity was low (I² = 4.5%, p = 0.38), supporting the robustness of the findings (Figure 4).

Subgroup analyses by individual agents revealed consistent trends across most SGLT2 inhibitors, although the magnitude of benefit varied. Empagliflozin (RR 0.65, 95% CI: 0.50-0.85, p = 0.001), canagliflozin (RR 0.61, 95% CI: 0.45-0.83, p = 0.002), dapagliflozin (RR 0.74, 95% CI: 0.62-0.88, p = 0.0008), and sotagliflozin (RR 0.69, 95% CI: 0.55-0.88, p = 0.002) were all associated with statistically significant reductions in HHF. In contrast, ertugliflozin (RR 0.85, 95% CI: 0.69-1.06, p = 0.15) showed a non-significant trend toward benefit, possibly reflecting differences in study design, patient population, or statistical power. As the confidence intervals for individual agents overlap, these results suggest that the observed reduction in HHF is likely a class effect of SGLT2 inhibitors rather than a drug-specific phenomenon. These findings collectively confirm that SGLT2 inhibitors significantly reduce the risk of HHF in patients with type 2 diabetes, with relatively consistent benefits across agents, except for ertugliflozin, where evidence remains less conclusive.

Secondary Outcomes

Although several included trials reported additional cardiovascular or renal outcomes (such as cardiovascular death, renal composite endpoints, and all-cause mortality), these were not consistently reported across all studies. Due to this heterogeneity, a quantitative synthesis of secondary endpoints was not feasible. Therefore, the present meta-analysis was restricted to the primary outcome of HHF.

Sensitivity Analyses

No formal sensitivity analyses, such as leave-one-out testing or comparison of fixed-effect versus random-effects models, were performed. This decision was based on the relatively small number of included studies and the low observed heterogeneity (I² = 4.5%). Given the consistency of findings across the majority of trials, the results are considered robust without additional sensitivity testing. Funnel plot analysis was not performed due to the limited number of included studies (n = 6), as funnel plot asymmetry is considered unreliable when fewer than 10 studies are available. Therefore, a formal assessment of publication bias was not conducted.

Discussion

Principal Findings

This meta-analysis of six large-scale RCTs, enrolling a combined total of 47,411 patients with T2DM, demonstrated that SGLT2 inhibitors significantly reduced the risk of HHF by 28% compared with placebo or standard care (RR: 0.72, 95% CI: 0.65-0.80; p<0.00001). The benefit was consistent across most individual agents. Empagliflozin (RR: 0.65, 95% CI: 0.50-0.85), canagliflozin (RR: 0.61, 95% CI: 0.45-0.83), dapagliflozin (RR: 0.74, 95% CI: 0.62-0.88), and sotagliflozin (RR: 0.69, 95% CI: 0.55-0.88) all demonstrated statistically significant reductions in HHF risk. Ertugliflozin showed a nonsignificant trend toward benefit (RR: 0.85, 95% CI: 0.69-1.06), likely reflecting lower event rates and statistical power in the VERTIS-CV trial [9]. Importantly, these findings were achieved despite heterogeneity in trial populations, including patients with established ASCVD, CKD, and multiple cardiovascular risk factors, underscoring the broad applicability of SGLT2 inhibitors across diverse high-risk subgroups.

Comparison With Prior Evidence

Our findings are consistent with, and extend upon, earlier CVOTs and meta-analyses that first established the cardiorenal benefits of SGLT2 inhibitors. The seminal EMPA-REG OUTCOME trial (2015) [5] demonstrated a 35% relative risk reduction in HHF with empagliflozin, while the CANVAS program (2017) [6] confirmed a 33% reduction with canagliflozin. DECLARE-TIMI 58 (2019) [7], the largest trial to date with over 17,000 participants, showed a 27% reduction in HHF despite including a lower-risk population in which only 40% had ASCVD. More recently, CREDENCE (2019) [8] highlighted canagliflozin’s efficacy in CKD, while SCORED (2021) [10] confirmed sotagliflozin’s benefit even in patients with advanced renal impairment, albeit with early termination. Our pooled estimate (RR: 0.72) aligns closely with Zelniker et al.’s Lancet 2019 meta-analysis [14] (RR: 0.69, 95% CI: 0.61-0.79) and some other significant studies [15] supporting SGLT2 inhibitors as a cornerstone therapy in HF prevention among T2DM patients. A key strength of our study is the strict inclusion of only RCTs with ≥12 months of follow-up, which reduces the risk of overestimating short-term effects and enhances the reliability of long-term outcomes. Taken together, our results reinforce and strengthen the evidence base that SGLT2 inhibitors confer robust and consistent reductions in HHF risk across diverse populations.

Clinical and Biological Interpretation

The observed reduction in hospitalization for heart failure with SGLT2 inhibitors can be explained by several synergistic mechanisms that extend beyond glucose lowering. By inducing osmotic diuresis and natriuresis, these agents reduce preload and ventricular wall stress without causing excessive intravascular volume depletion, thereby minimizing the risk of hypotension compared to traditional diuretics [16]. Importantly, SGLT2 inhibitors preferentially reduce interstitial fluid rather than plasma volume, a distinction that alleviates congestion while preserving hemodynamic stability. Experimental data also suggest favorable effects on myocardial energetics, with shifts toward ketone utilization that enhance cardiac efficiency and reverse maladaptive remodeling [17,18]. In parallel, renoprotective actions - mediated by reduced intraglomerular pressure and preservation of nephron integrity - help attenuate cardiorenal syndrome, a critical driver of HF progression. Collectively, these mechanisms emphasize that the cardioprotective benefits of SGLT2 inhibitors are largely independent of glycemic control, highlighting their role as disease-modifying therapies in T2DM patients at high cardiovascular risk [19,20].

Clinical Implications

Our findings provide strong support for current guideline recommendations by the American Diabetes Association (ADA), European Society of Cardiology (ESC), and Kidney Disease: Improving Global Outcomes (KDIGO), all of which endorse SGLT2 inhibitors as first-line therapy in T2DM patients with heart failure or established cardiovascular disease [21,22]. Importantly, the consistent reduction in HHF observed across both ASCVD-focused trials (EMPA-REG [5], VERTIS CV [9]) and renal-focused trials (CREDENCE [8], SCORED [10]) underscores their broad applicability across diverse patient populations. The inclusion of lower-risk patients in DECLARE-TIMI 58 [7], where significant benefit was still demonstrated, further suggests potential utility in primary prevention of HF hospitalization among individuals without established CVD. Taken together, these results reinforce the positioning of SGLT2 inhibitors not merely as glucose-lowering drugs, but as cornerstone therapies in the comprehensive management of cardiometabolic disease.

Limitations

Several limitations of this study should be acknowledged. First, the evidence base is limited to six RCTs, restricting the power of subgroup and sensitivity analyses. Second, there was heterogeneity in patient populations across trials, with some focusing exclusively on ASCVD, others on CKD, and still others enrolling mixed-risk cohorts. Follow-up duration varied substantially, ranging from 1.3 years in SCORED [10] to over four years in DECLARE-TIMI 58 [7], which may have influenced event accrual and effect estimates. Additionally, early termination of the SCORED [10] trial raises concerns about potential bias and incomplete capture of long-term outcomes. We were unable to pool secondary endpoints such as cardiovascular death and renal outcomes due to heterogeneity in reporting, and the small number of included trials limits the reliability of publication bias assessment. These factors should be considered when interpreting the results.

Future directions

Future research should focus on addressing remaining knowledge gaps in the role of SGLT2 inhibitors for HF prevention. Direct head-to-head comparisons between different agents are needed to determine whether efficacy is a true class effect or varies across molecules. Trials enrolling lower-risk T2DM patients without established ASCVD or CKD would clarify the utility of SGLT2 inhibitors in broader primary prevention. Mechanistic investigations are also warranted to disentangle differential effects on heart failure phenotypes, particularly heart failure with preserved ejection fraction (HFpEF) versus reduced ejection fraction (HFrEF) [23]. Moreover, combination therapy with GLP-1 receptor agonists holds promise for synergistic cardiovascular and renal protection, but requires robust evaluation in adequately powered RCTs. Such efforts will refine patient selection and therapeutic strategies, ultimately optimizing the cardiorenal outcomes of individuals with diabetes.

## Conclusions

This meta-analysis of six landmark RCTs, encompassing over 47,000 patients with type 2 diabetes, provides robust evidence that SGLT2 inhibitors significantly reduce the risk of HHF by nearly one-third compared with placebo or standard care. The consistency of benefit across diverse populations - including those with established ASCVD, CKD, and broader cardiometabolic risk - highlights the generalizability of these findings and reinforces the paradigm shift in diabetes care from purely glucose-centric management to cardiorenal risk reduction. By demonstrating clinically meaningful, durable benefits independent of glycemic control, our work underscores the role of SGLT2 inhibitors as cornerstone therapy for preventing heart failure in T2DM. These results strengthen guideline recommendations and support the widespread adoption of SGLT2 inhibitors in routine practice, with the potential to reduce the global burden of HF among patients with diabetes.
